# Peripheral Blood Lymphocytosis Reflecting an Underlying Thymoma

**DOI:** 10.1002/ajh.70437

**Published:** 2026-07-03

**Authors:** Léa Ousset, Alban Canali, Barbara J. Bain

**Affiliations:** ^1^ Laboratoire d'Hématologie Université de Toulouse, Centre Hospitalo‐universitaire (CHU) de Toulouse, Institut Universitaire du Cancer de Toulouse‐Oncopole (IUCT‐O) Toulouse France; ^2^ Centre for Haematology St Mary's Hospital Campus of Imperial College London Faculty of Medicine London UK

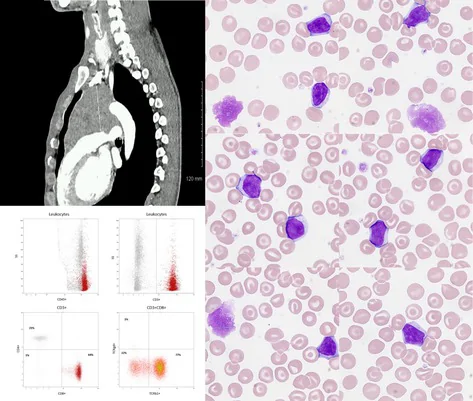

A 36‐year‐old man with well‐controlled asymptomatic myasthenia gravis presented for a biopsy of a 9 cm unresectable anterior mediastinal mass infiltrating the aortopulmonary window (top left, computed tomography). A blood count showed lymphocytosis (lymphocyte count 5.7 × 10^9^/L) and microcytosis without anemia (mean cell volume 74.5 fl), hemoglobin concentration 148 g/L (the patient was known to have α thalassemia trait). The blood film showed target cells and a monomorphic lymphocyte population composed of medium‐sized cells with a high nucleocytoplasmic ratio, a somewhat irregular nucleus with delicate chromatin, and weakly basophilic cytoplasm which was sometimes vacuolated; some smudge cells were present and nucleoli were apparent in these (right, composite panel, May‐Grünwald‐Giemsa, ×100 objective). The findings were suggestive of a low‐grade chronic lymphoproliferative neoplasm. Histopathological examination and immunohistochemistry of the biopsy specimen revealed a nodular lymphoid proliferation composed of numerous immature T cells (terminal deoxynucleotidyl transferase +/CD3+), with well‐defined medullary areas with clusters of B cells (CD20+/PAX5+), and a network of p40+ epithelial cells within and around the nodules, present either as isolated cells or in clusters of more than three, confirming the diagnosis of thymoma. Flow cytometry immunophenotyping from peripheral blood (bottom left) showed a CD3+ CD8+ lymphocyte subpopulation representing 64% of all lymphoid cells, without activation markers (CD16‐/CD56‐/CD57‐/HLA‐DR‐) and associated with polytypic expression of T‐cell receptor TRBC1 (77%), consistent with a CD8+ peripheral blood T‐lymphocytosis. Subsequently, positron emission tomography revealed two pleural masses indicating metastasis of the thymoma.

Peripheral blood T‐cell lymphocytosis is a rare and poorly understood autoimmune/paraneoplastic manifestation of thymoma, with fewer than 25 cases reported to date [1], and may represent the initial clue to this underlying entity. An abnormal peripheral blood film should not limit the differential diagnosis to hematologic malignancies alone and must be interpreted in the context of the clinical presentation. In this setting, assessment of morphological features combined with flow cytometric immunophenotyping is useful for avoiding misdiagnosis of lymphoproliferative neoplasms and establishing this rare but important diagnosis.

## Conflicts of Interest

The authors declare no conflicts of interest.

## Data Availability

The data that support the findings of this study are available from the corresponding author upon reasonable request.
